# Family Caregivers’ Self-Efficacy and Its Relations to Well-Being: Gender and Age Differences

**DOI:** 10.1093/geroni/igaa057.490

**Published:** 2020-12-16

**Authors:** Yu-Ping Chang, Young Seo, Tania Von Visger

**Affiliations:** University at Buffalo, Buffalo, New York, United States

## Abstract

Family caregivers of older adults perceive their roles as beneficial as well as burdensome. They also report physical and psychological symptoms affecting their well-being. Research indicates that caregiver self-efficacy leads to positive outcomes such as decreased caregiver burden and stress. However, it is unclear how self-efficacy is related to their well-being, and how this association might differ by age and gender. We aim to explore the relationships between family caregiver self-efficacy and well-being, and determine if age or gender moderates these relationships. We analyzed responses of 2,652 family caregivers (68% female and 32% male; mean age= 61.2) from the National Study of Caregiving (NSOC) III (2017) cross-sectional survey. We used three composite variables measuring self-efficacy: feeling confident, adjusting changes, and recovering quickly. We used five composite variables measuring well-being: pain, sleep problems, positive and negative affects, and depression/anxiety. We conducted a design-based weighted logistic regression analysis to examine the relationships among variables of interest. Results indicated that low self-efficacy was associated with increased pain, sleep problems, positive and negative affects, controlling for caregivers’ age, sex, and race/ethnicity. Examination of interactions indicated that the relationship between self-efficacy and pain and the relationship between self-efficacy and depression/anxiety were stronger in female caregivers, whereas age did not serve as a moderator in the relationship between self-efficacy and well-being. Our findings provide insight that can guide intervention development to improve family caregiver self-efficacy and well-being. Furthermore, future research may involve interventions with the consideration of family caregivers’ gender.

